# High‐dose rate intracavitary brachytherapy pretreatment dwell position verification using a transparent applicator

**DOI:** 10.1002/acm2.12405

**Published:** 2018-06-30

**Authors:** Yuki Otani, Iori Sumida, Takayuki Nose, Shigetoshi Shimamoto, Hirofumi Okubo, Kazuhiko Ogawa

**Affiliations:** ^1^ Department of Radiology Kaizuka City Hospital Osaka Japan; ^2^ Department of Radiation Oncology Osaka University Graduate School of Medicine Osaka Japan; ^3^ Department of Radiation Oncology Nippon Medical School Tama Nagayama Hospital Tokyo Japan; ^4^ Department of Radiation Oncology Osaka General Medical Center Osaka Japan; ^5^ Department of Radiation Oncology Sakai City Medical Center Osaka Japan

**Keywords:** 3D printing, brachytherapy, transparent applicator, verification

## Abstract

**Purpose:**

The major errors in HDR brachytherapy are related to treatment distance, almost all of which are caused by incorrect applicator information. The aim of this study is to propose a quick pretreatment verification method to evaluate channel length and dwell position with a transparent applicator, which, in addition, is suitable as an education tool to assist in the understanding of the applicator structure.

**Methods:**

A transparent applicator model was fabricated using a three‐dimensional printer and transparent resin. Its aim is to be a replica of a real gynecological applicator. The pretreatment verification is performed by observing the planned dwell positions of a check cable inside a transparent applicator. A digital camera acquired images and the dwell positions of the radioactive source and check cable were evaluated by comparing them with respect to the theoretical dwell positions marked by the proper x‐ray marker. The potential effectiveness of verification using a transparent applicator was also evaluated using brachytherapy events reported in the literature.

**Results:**

The transparent applicator closely resembles the real applicator in shape and had an error of less than 0.2 mm. The average dwell position displacement between the radioactive source and check cable was 0.4 mm. The analysis of brachytherapy events showed that channel‐length, dwell‐position, and step‐size errors made up 50% of all events, but affected 64% of all patients.

**Conclusions:**

The transparent applicator model enables a noninvasive, repeatable verification of the channel length and dwell positions to be performed before treatment. This verification has the potential to help prevent common errors in treatment delivery. In addition, the transparent applicator model can be used as a teaching tool to help clinicians understand the operation of the applicator, lowering the risk of events.

## 1 INTRODUCTION

In high‐dose rate (HDR) brachytherapy for cervical cancer, an applicator is placed in the vagina and penetrates the cervical canal.^1^ One of several types of applicators is chosen based on the expansion of the cancer and patient's status.^2^ In treatment planning, it is essential to input the correct applicator information (i.e., the offset value and channel length). However, the correct relationship between the first dwell position, offset value, and channel length can be hard to understand because each value depends not only on applicator type but also on the applicator reconstruction method in treatment planning.^3^ Because applicators are opaque, users cannot directly visualize the source path inside it with the naked eye, which is one reason incorrect applicator information may be considered. Consequently, incorrect applicator information may result in irradiation at a position that differs from the treatment plan, which might cause unexpected adverse events and the expected therapeutic effects may not be obtained.

Several radiation misdeliveries caused by incorrect applicator information have been reported, all attributable to a lack of understanding of the applicator structure.^4^, ^5^, ^6^, ^7^, ^8^, ^9^, ^10^, ^11^, ^12^, ^13^ To prevent accidents, independent pretreatment verification is important. However, the common source position check ruler can be limited because it is generally restricted for use with a specific transfer tube type.^14^ If the planned dwell position of the source inside an applicator can be observed, it can be compared with the dwell position displayed on the digitally reconstructed applicator in the treatment plan. An unintended dwell position will be instantly noticed based on an intuitive estimation. Therefore, this study aims to propose a quick pretreatment verification method to evaluate channel length and source dwell position with a transparent applicator and, in addition, suggest its use as an educational instrument.

## 2 MATERIALS AND METHODS

### 2.A Transparent applicator fabrication

**Figure 1:**
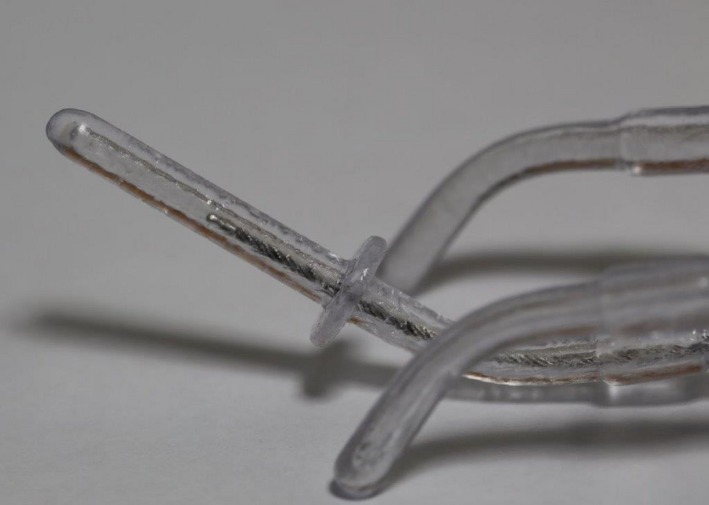
Appearance of a transparent applicator model of the Fletcher CT/MR applicator.

**Figure 2:**
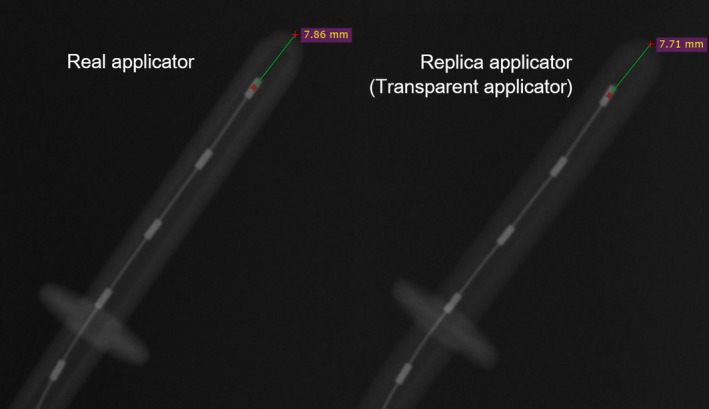
Autoradiographic comparison of the shape of the real tandem applicator and a transparent applicator.

To fabricate a transparent applicator, we chose to prepare a Fletcher CT/MR applicator (Nucletron, Elekta AB, Stockholm, Sweden), comprising a tandem and ovoids, which is used in intracavitary brachytherapy (ICBT) for cervical cancer. A commercially available Fletcher‐type applicator was scanned using computed tomography (CT; Aquilion LB, Toshiba medical systems, Tochigi, Japan), and three‐dimensional (3D) data were acquired. The CT data were acquired in the axial cine mode. The slice thickness was 0.5 mm, which was reconstructed in a field of view of 240 mm on a 512 × 512 grid. All CT slices were transferred to a Digital Imaging and Communication in Medicine (DICOM) viewer (OsiriX Lite version 7.0.3, Bernex, Switzerland) to perform surface rendering. The file format was then changed from DICOM to standard triangulation language (STL). The junction of the applicator and the transfer tube could not be accurately acquired from the CT data because the connector region structure was rather thin. The vendor (Nucletron, Elekta AB, Stockholm, Sweden) hence provided the design of the applicator after we signed a nondisclosure agreement. Based on this design, the 3D data of the connector region were modified using computer graphics software (Shade 3D version 15, Shade3D, Tokyo, Japan). The transparent applicators were fabricated using a 3D printer (ProX800; SOLIZE Products, Kanagawa, Japan) with a minimum layer height of 50 μm and accuracy of 0.05 mm using epoxy polypropylene‐based resin as the printing material (Fig. 1). After sculpting a transparent applicator using the 3D printer, the manufacturing accuracy was confirmed by autoradiography (Fig. 2).

### 2.B Evaluation of the practicality and accuracy of verification using a transparent applicator

**Figure 3:**
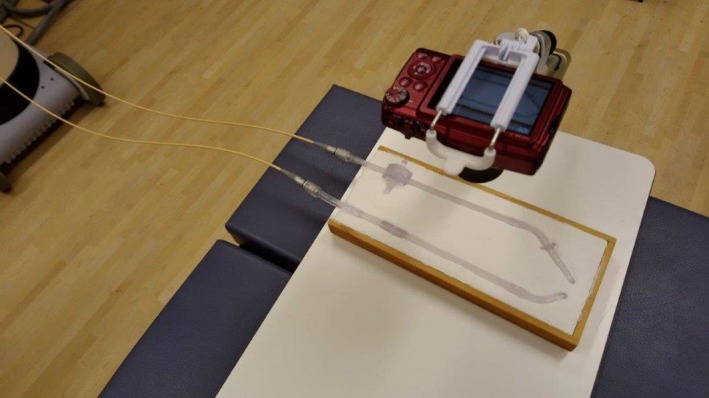
Image‐based measurement setup.

**Figure 4:**
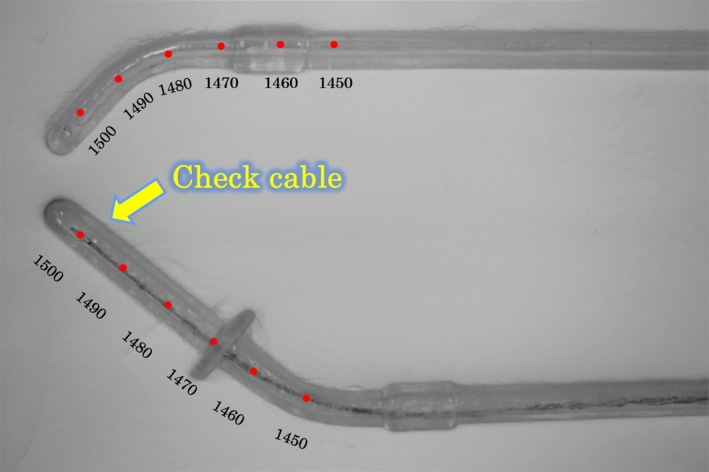
Red circles show the dwell positions determined by the x‐ray marker.

In this study, the verification was performed with a microSelectron‐HDR V2 (version 1.51) and Oncentra (version 10; Nucletron, Elekta AB, Stockholm, Sweden). First, an x‐ray marker was inserted into the transparent applicators (right ovoid and tandem), and a picture was taken (Fig. 3). The position recognition scale marks were provided digitally based on the proper x‐ray marker at 10‐mm intervals from 1500 to 1450 mm (Fig. 4). Next, the transparent applicator was connected to the HDR unit through a transfer tube and the check cable and radioactive source run was conducted at 10‐mm intervals from 1500 to 1450 mm (with a step size of 2.5 mm). In this verification, the check cable position test mode was used, and the setting of each check cable dwell time was 1 s. To evaluate the reproducibility and mechanical variation, the check cable and radioactive source run were conducted five times each. For this verification, the preestablished dwell positions in a quality assurance (QA) plan was used.

A digital camera acquired images at 30 frames/s with a 1920 × 1080 pixel resolution and 0.07 mm/pixel. The radioactive source and check cable position were estimated using distance measuring software (Acrobat 9 Pro; Adobe Systems, CA, USA). The viewing angle of the picture was corrected using software (Photoshop Elements; Adobe Systems, CA, USA), and the position error was calculated from the scale marks to the center of the source capsule.

### 2.C Pretreatment verification methodology

The verification is performed by observing the dwell positions of a check cable with a transparent applicator. The check cable is a standard feature in commercial HDR treatment machines and is a dummy source that confirms the pathway before the radioactive source is driven out. First, the isomorphic transparent applicator model of an intracavitary applicator in a patient is prepared. After treatment planning, the transparent applicator is connected to the HDR unit through a transfer tube and the check cable run is verified. After confirming the dwell position of the check cable, it is switched with a transfer tube connecting to the indwelling applicator in the patient.

In the microSelectron‐HDR, there are two operation modes of the check cable. One is the extra check cable run mode: the check cable overshoots the most distal dwell position of the radioactive source by 5 mm. It then retracts to this point and dwells there for 1 s. The other mode is the check cable position test mode; the check cable dwells at each dwell position of the radioactive source (the same positions in versions 1.3, 1.4, and 1.5 of the microSelectron‐HDR. The check cable dwell position is 2 mm longer than the radioactive source dwell positions in versions 3.1.3.800, 3.1.4, and 3.1.5.500).

The initial setting of each check cable dwell time is 1 s, but this time can be changed as required.

### 2.D Previous HDR event collection

To assess the potential effectiveness of verification using a transparent applicator, we searched the literature for previous studies reporting HDR events and analyzed them. We used information concerning incidents from the International Commission on Radiological Protection (ICRP) and the Nuclear Regulatory Commission (NRC) summarized by Nose et al.^5^, ^6^, ^15^ We reorganized table 4 from Nose et al. and added the number of patients. Moreover, we analyzed information from the microSelectron‐HDR user community site (UCS), which records details of events in Japan and is managed by Chiyoda Technol Corporation, Nucletron's Japanese distributor. The UCS reported nine events that occurred from 2008 to 2017.^4^

## 3 RESULTS

### 3.A Evaluation of the practicality and accuracy of verification using a transparent applicator

**Figure 5:**
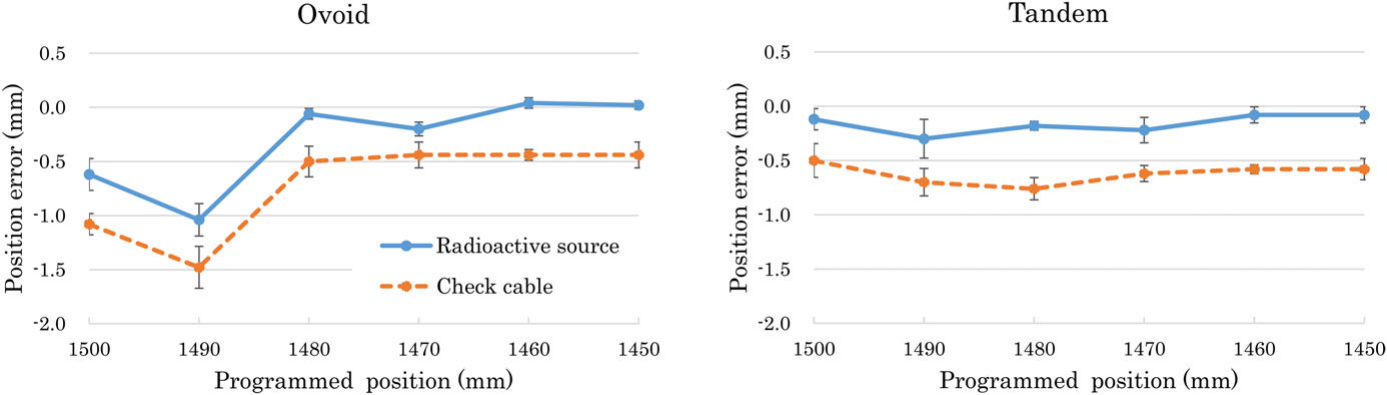
Difference between the programmed and measured positions of the radioactive source and check cable. A negative value indicates that the measured position is shorter than programmed position. The graphs show the mean (±1*σ*) at each dwell position.

The Fletcher‐type applicator, fabricated using epoxy polypropylene‐based resin, was transparent, allowing a clear observation of the inside of the applicator. The transparent applicator closely resembles the real applicator in shape and had an error less than 0.2 mm (Fig. 2). Figure 5 plots the difference between the programmed and measured positions of the radioactive source and check cable. A negative value indicates that the dwell position was shorter than the programmed position. In the ovoid applicator, the maximum dwell position displacements were −1.2 and −1.8 mm for the radioactive source and check cable, respectively. In the tandem applicator, the maximum dwell position displacements were −0.5 and −0.9 mm for the radioactive source and check cable, respectively. A systematic displacement was observed in the dwell position of the radioactive source and check cable. The dwell position of the check cable was shorter than the radioactive source, and the mean and standard deviation of the dwell position difference between the check cable and radioactive source were −0.4 ± 0.2 mm.

### 3.B Previous HDR event collection

Table 1 shows 54 events that were categorized as dwell position‐related, dwell time‐related, and dwell‐position/time‐unrelated events. Of these, the wrong channel length (22%) was the most common error, followed by the wrong dwell position (20%). For three subcategories (i.e., wrong channel length, wrong dwell position, and wrong step size), one event involved several patients, implying that these events were not noticed for several treatments. Moreover, the same error was repeated in multiple patients. Consequently, these three subcategories made up 50% of all events, but affected 64% of all patients.

**Table 1:** Categories of 54 HDR treatment events from ICRP 97 and NRC (Jan 2007 to Sept 2011).^5^, ^6^, ^15^

Category	Subcategory	Events, *n* (%)	Number of patient, *n* (%)	Detectability of transparent applicator
Dwell position‐related events	Wrong channel length	12 (22)	22 (30)	Yes
	Wrong dwell position	11 (20)^a^	13 (18)	Yes
	Wrong step size	4 (7)	12 (16)	Yes
	Wrong applicator position/setting	9 (17)	9 (12)	
	Wrong applicator connection	2 (4)	2 (3)	
	Other	4 (7)	4 (5)	
Dwell time‐related events	Wrong prescription dose	7 (13)^a^	7 (9)	
	Other	4 (7)	4 (5)	
Dwell position/time‐nonrelated events	Irradiation without connecting transfer tube	1 (2)	1 (1)	
	Accidental pushing of auto radiography button vs treatment button	1 (2)	1 (1)	
	Source contamination by blood	1 (2)	1 (1)	
Total		54	74	

HDR, high‐dose‐rate; ICRP, International Commission on Radiological Protection; NRC, Nuclear Regulatory Commission.

a Two events overlapping with dwell position related and dwell time related.

Furthermore, we analyzed the theoretical detectability of the events using our approach. There are only three subcategories of events (i.e., wrong channel length, wrong dwell position, and wrong step size) that could be detected using a transparent applicator; however, these types of errors frequently occur.

Table 2 shows the nine events reported from microSelectron‐HDR UCS in Japan. While one event was for interstitial brachytherapy (inverse catheter direction), the others were ICBT errors. The wrong channel length and dwell position events made up 40% of all events. Notably, there were two events caused by time lag. One event was caused by an error in the computer system time: the treatment console system (TCS) time was 2 h earlier than the actual time. In the other one, the irradiation time was reduced to one eighth the intended time because of compatibility problems between the TCS and treatment planning system.

**Table 2:** Categories of nine HDR treatment events from the microSelectron‐HDR user community site in Japan (March 2008 to March 2017).^4^

Category	Subcategory	Detail	Events, *n* (%)	Number of patients, *n* (%)
Dwell position‐related events	Wrong channel length	Inputting channel length, 5 mm too long	1 (11)	1 (11)
	Wrong dwell position	Inversed catheter direction	2 (22)	2 (22)
		Inputting off set value, 6 mm too short	1 (11)	1 (11)
		Patient identification	1 (11)^a^	1 (11)^a^
Dwell time‐related events	Wrong prescription dose	Inputting cylinder diameter, 5 mm too small	1 (11)	1 (11)
		Malfunction, the time lag	2 (22)	2 (22)
		Patient identification	1 (11)^a^	1 (11)^a^
Dwell position/time‐nonrelated events	Other	Malfunction, the source does not return	1 (11)	1 (11)
Total			9	9

a One event overlapping with dwell position related and dwell time related.

## 4 DISCUSSION

In this study, we proposed a novel, simple, quick, and noninvasive pretreatment verification method that uses the check cable and a transparent applicator. Although we used a camera for quantitative evaluation in this study, in clinical practice, visual inspection alone would be sufficient. Any position discrepancy of over 2 mm can be noticed immediately, and this verification can be repeated as many times as needed. The time taken for a series of verification flows was about 2 min, which is a reasonable time for patients as well as medical staff. A time‐consuming verification method should be avoided because this may increase the potential treatment delivery error caused by geometric changes that occur between planning and treatment delivery. Large dwell position displacements were observed at the curved portion of the ovoid applicator (Fig. 5), which is larger than the required 1 mm source position accuracy.^16^ The cause of that the pathways are different between the x‐ray marker and wire (radioactive source and check cable).^17^ In this study, the transparent applicator was fabricated using a 3D printer to demonstrate its concept and practicality. The intent is not to recommend the creation of applicators using 3D printers. For clinical use worldwide, it will be necessary to create applicators using molds, and the vendor should produce a transparent applicator as an official product.

Table 1 shows that events were frequently caused by the wrong channel length, wrong dwell position, and wrong step size. These events have been reported to occur in several patients per event. In other words, the detection of these three events is essential for avoiding large‐scale accidents. In December 2013, a systematic misdelivery involving as many as 100 patients was disclosed in Japan, all of which occurred because of a lack of independent verification methods before treatment delivery.^12^, ^13^ This accident was caused by a misunderstanding of the channel length and consisted of the systematic insertion of a 3‐cm shorter length for ovoid applicators. Unfortunately, this systematic error remained unnoticed by personnel for 7 yr (2007–2013). Considering these accidents, we designed a verification method that focuses on the channel length and dwell position. This verification method can be used not only for the microSelectron‐HDR but also for the VariSource (Varian Medical Systems, California, USA). Note that the check cable dwells only at the most distal dwell position of the radioactive source in a VariSource.

An institution that has a C‐arm may monitor the check cable in the indwelling applicator in the patient using a fluoroscope.^18^ Nose et al. reported a real‐time verification method of radioactive source position using modified C‐arm fluoroscopy.^15^ Although it is ideal to monitor the radioactive source during treatment, radiation regulations may restrict the use of this method. In some countries, regulations limit the concurrent use of two independent radiation sources in a single suite.^19^, ^20^

Researchers have summarized the errors and variations in brachytherapy along with their potential for detection by each verification method.^21^, ^22^ Not all events can be detected by a single verification method. Moreover, detectability varies depending on the event type. To increase the detection rate of the proposed approach, combining verification methods would be effective. For example, Das et al. proposed an easy verification method for checking the dwell time that uses the relationships among prescription dose, source activity, and irradiation volume.^23^ A combination of verification methods would compensate for each method's shortcomings and enhance the detectability of treatment planning error.

The limitation of the current approach is that systematic differences may exist between the delivery conditions in the real applicator and a transparent applicator. Moreover, the stop position of the check cable and radioactive source may be different because each are driven by different mechanical drive motors. In our results, the dwell position difference between the check cable and the radioactive source was less than 1 mm (Fig. 5). In addition, the check cable is calibrated to the same tolerance as the radioactive source (within ±1 mm of nominal). However, the frequency of the check cable driver is about twice that of the radioactive source, and fatigue and skew can occur in the cable. Therefore, a periodic QA procedure to verify the stop position of the check cable and a commissioning of the transparent applicator is mandatory before verification.

Transparent applicators will be useful as training tools. Some researchers have reported that failure modes in HDR brachytherapy tend to occur because of human error during treatment planning and delivery rather than because of malfunctions of the treatment machine.^10^, ^11^, ^24^ Failures frequently result because of lack of training or experience. Proper equipment commissioning and sufficient training are key for avoiding accidents. Training using transparent applicators will make the relationship between the x‐ray marker position, radioactive source position, channel length, and offset easy to understand. Wang et al. provided a good tutorial on applicator reconstruction in the treatment planning process.^3^ The use of transparent applicators will enable more effective training and assist understanding of the applicator structure.

The current transparent applicator was only designed for pretreatment verification, and the purpose of this verification is to catch gross channel‐length, dwell‐position, and step‐size errors. Note that this verification is not meant to replace the commissioning and periodic QA of real applicators, which require millimeter accuracy because the transparent applicator is a replica and not available for use on patients yet. To use it with patients, it will be necessary to find a biocompatible material that meets the durability and sterilization requirements of applicators. If this problem can be solved, all applicators used for the patient can be transparent, which may be advantageous for QA. The evaluation of the source position is a crucial QA practice in HDR brachytherapy, and these procedures are laborious. A transparent applicator would make it possible to evaluate the source position visually.

## 5 CONCLUSIONS

A transparent applicator will be a good educational tool for improving the understanding of the applicator structure. This implementation of the pretreatment verification method is a simple, quick, and noninvasive, and it enables instantaneous detection of dwell position error that can be estimated visually. This method not only detects dwell position errors, but also errors in the input of the applicator's geometrical parameters and step size. Any significant difference between the actual and intended dwell position of the check cable can be easily identified during verification, thus lowering the risk of fatal errors.

## CONFLICTS OF INTEREST

The authors declare no conflicts of interest associated with this manuscript.
